# Content analysis of vitamins, dietary fibers and amino acids in a wide collection of barley (Hordeum vulgare L.) from Tibet, China

**DOI:** 10.6026/97320630016314

**Published:** 2020-04-30

**Authors:** Haijiao Huang, Xiaoli Gao, Yang Li, Pengjia Tian, Yangzong Nima, Zhaxi Laba, Zhen Ci, Xinhong Wei, Ji Qu, Weixing Guan, Wenhua Liao

**Affiliations:** 1 Tibet Academy of Agricultural and Animal Husbandry Sciences, Lhasa 850000, China

**Keywords:** nutritional diversity, germplasm, barley, China, crop improvement

## Abstract

Barley (Hordeum vulgare L.) is an important agricultural crop. Various studies on the genetic diversity, biochemical and molecular attributes on this species are known. However, information on nutritional variability in a large panel of barley cultivars is limited. Therefore, it is of interest for a quantitative analysis of vitamins, amino acids and dietary fibers in 245 barley of Tibet region in China. The coefficient of variation analysis revealed strong variation of vitamins (VB1>VB2>VE), essential amino acids (valine, histidine, methionine, lysine), non-essential amino acids (proline, tyrosine, cysteine), dietary fibers, (cellulose > lignin). Principal component analysis detected three clusters of cultivars, each with specific characteristics. However, the most nutritional cultivars were found in Cluster 3, which encompassed 52 cultivars. Distinctly, six cultivars (ZQ2000, BJX230, BJX229, BJX249, BJX191 and BJX265) were identified with highest nutritional values. This study reveals a large nutritional diversity in barley cultivars from Tibet and represents an important reference for the exploitation of these germplasm in crop improvement and breeding programmes.

## Background

Barley (Hordeum vulgare L.) belongs to the Poaceae family. It is the fourth important cereal crop cultivated around the world after wheat, rice; maize in terms of quantity produced and planted areas [1]. Its cultivated areas are mainly located in Europe (Poland, Finland, England, and Denmark), Asia (Iran, Japan, India, Korea and Tibet region of China) and Africa (from Northern to Horn Africa) [2]. Its origin of diversity is not well known yet but the South-Eastern Asia region, including China, Tibet, and Nepal has been proposed [3]. The second centre of diversity is thought to be in Ethiopia [4]. The world production of barley in 2018 amounted to 142.37 million metric tons [5]. The barley production of Tibet was estimated in 2017 at 700,000 tons harvested from 0.14 million hectares [6]. This represents 70% of total highland barley production of China. Barley is widely used for human consumption and also for animal feeding. It is widely used in brewing and bakery industries for malt production mainly in brewing beer [7] and is processed into barley vinegar, biscuits, malted food drinks and sugar confectionery [8].

Owing to its nutritional and bioactive compounds, barley is highly desired for its health benefits. Its flour is reported to contain 54.2% of starch, 2.42% of crude fat, 2.92% of total ash, 14.2% of protein, 2.86% of soluble dietary fiber, 10.24% of insoluble dietary fiber and 13.1% of total dietary fiber [9]. The regular consumption of fiber-rich foods is shown to be associated with the reduction of the cardiovascular diseases (coronary heart disease, stroke and hypertension) and in the prevention of colon cancer [9-11]. Many studies mentioned its ability to improve human health by lowering cholesterol and sugar levels in blood and thus preventing cardiovascular diseases and diabetes [12,13]. Barley is also enriched in essential amino acids making it a suitable food to meet the requirements for human body [14]. Compared to other cereal crops, barley has higher content in lysine, important amino acid involved in the development of muscle tissue in animals, especially for the pig farming [15]. Moreover, barley is source of functional compounds like vitamins B1, B2 and B3 and also vitamin E, essential to preserve the nervous system, and prevent skin diseases, diabetes and some cancers [16,17].

Many studies have addressed the genetic diversity of barley through morphological and molecular criteria mainly for the purpose of domestication, conservation and genetic improvement of the crop [18-23]. However, few have focused on the diversity based on nutritional properties in a wide barley collection. The growing demand for functional foods with high health benefits urges us to explore the existing diversity to identify cultivars of high nutritional values [24, 25]. The aim of the present study was to assess the variability of vitamins, dietary fibers and amino acids contents in a wide collection of barley (Hordeum vulgare L.) collected in Tibet region of China. The findings will inform on relationships between nutritional traits and promising cultivars useful for breeding programmes aiming at improving the nutritional and functional properties of barley seeds.

## Material and Methods:

### Plant materials:

Two hundred forty five (245) barley (Hordeum vulgare L.) cultivars were collected in 12 different counties of Tibet and preserved at the Tibet Academy of Agriculture and Animal Husbandry Sciences. Details on the collection are presented in (Table S1). Seeds were sown in pots containing peat and plants were maintained in a greenhouse at the plant science department of the Tibet Academy of Agriculture and Animal Husbandry Sciences.

### Biochemical analysis:

The seeds were harvested from the 245 cultivars and were used to determine the content of vitamins, dietary fibers and amino acids. The methods used to perform these analyses were previously fully described. Vitamins B1, B2, B3 and E were detected following procedures described by Miranda et al. [26]. The lignin content in the barley grain was performed as described by (Kirk and Obst [27]. The cellulose content was determined using a spectrophotometer as described by Shet et al. [28]. The amino acids extraction and analysis were done as described by Choi et al. [29].

### Data analysis:

Descriptive parameters such as mean, coefficient of variation and range were determined on the nutritional compounds to describe their variability within the studied collection. Pearson coefficient of correlation was determined to estimate the correlation between the nutritional parameters; a correlogram was drawn using the corrplot package. To deepen the relationships among the nutritional variables and study the structure of the collection, a hierarchical cluster analysis on principal components was performed using the FactoMineR package. Analysis o variance followed by the mean comparison test of Tukey was then performed to compare the different clusters obtained. All the data analysis was performed using R version 3.6.2.

## Results

### Variation of the contents of vitamins, dietary fibers and amino acids within the Tibetan barley collection:

(Table 1) shows the variation of vitamins, γ-aminobutyric acid (GABA) and dietary fibers within the Tibetan barley collection. Based on the coefficient of variation, which relates the dispersion of a trait around its mean, and thus its level of variability, we found that vitamins B1 and B2 were the most variable within this collection. VB1 was in the highest proportion followed by VB2 and VE while VB3 was in the lowest proportion in the collection. The content in GABA was also highly variable. As for the dietary fibers, the content of cellulose was more variable and was in average, highly contained in these cultivars than lignin.

Essential amino acids represent in average 28% of total amino acids versus 72% for non-essential amino acids (Table 2).Among the essential amino acids, valine, histidine, methionine, lysine were the most variable. The highest contents were observed for leucine, phenylalanine, threonine and valine while methionine and valine were in the lowest proportions. As for the non-essential amino acids, proline, tyrosine, cystine were the most variable. This collection was particularly endowed in glutamic acid. This was followed by aspartic acid and proline while Tyrosine was in the lowest proportion.

### Correlations between the vitamins, dietary fiber and amino-acids:

**Figure 1:**
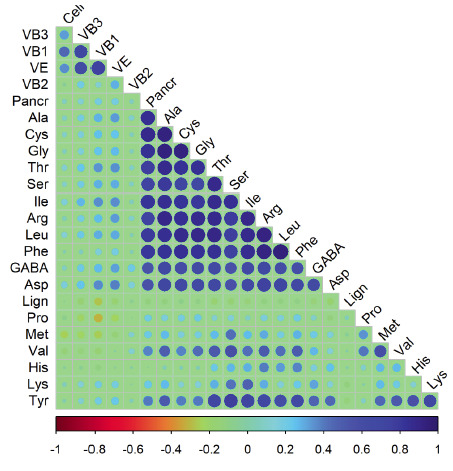
Pearson's correlation matrix for vitamin, dietary fiber and amino acids. The circles showed significant data at p < 0.05 (where VB1, vitamin B1 ; VB2, vitamin B2 ; VB3, vitamin B3 ;VE, vitamin E ; GABA= gamma-aminobutyric acid ; Cell, cellulose ; Lign, lignin ; Pro, proline ; Lys, lysine ; Leu, leucine ; Ile, isoleucine ; Phe, phenylalanine ; Met, methionine ; Val, valine ; Cys, cystine ; Tyr, tyrosine ; Ala, alanine ; Arg, arginine ; Thr, threonine ; Gly, glycine ; His, histidine ; Ser, serine ; Glu, glutamic acid ; and Asp, aspartic acid).

Significant correlations were found between many vitamins, dietary fibers and amino acids (Figure 1,Table S2). Essential amino acids such as Leu and Ile, and the non-essential amino acids as Glu, Ala, Cys, Gly, Thr, Ser, Arg, Phe, Asp and GABA were strongly pair-wisely correlated among each other. Similarly, Val and Tyr were also strongly correlated to Glu, Ala, Cys, Gly, Thr, Ser, Ile, Arg, Leu, Phe and GABA. Moreover, cellulose and the vitamins VB3, VB1 and VBE showed strong correlations among each other. However, lignin was negatively correlated to Ile, Tyr, Ser and Asp.

### Principal component analysis based on the vitamins, dietary fibers and amino acids:

**Figure 2:**
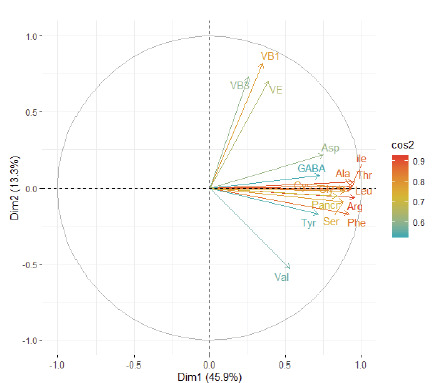
Relationship between the studied variables based on principal component analysis; only variables significantly represented on the principal components (cos2 > 0.5) are displayed.

Twenty four principal components explained the overall variability in nutritional parameters of the entire collection. The first five of these components reported in (Table 3), revealed 76.6% of the total variability. The first component (Dim.1 axis) described 45.9% of the total variability while the second (Dim.2) accounted for 13.3%. Out of the 24 studied variables, based on their cos2 values, 17 most discriminating variables were identified and ploted on (Figure 2). The first axis (Dim.1) mainly contributed to the variability of Arg, Ile, Thr, Ala, Leu, Phe, Gly, Cys, Ser, Glu, Asp, GABA, Tyr, while the second axis (Dim.2) explained the variability of VB1, VB3, VE (Table S3).

### Clusters of cultivars and their characteristics:

**Figure 3:**
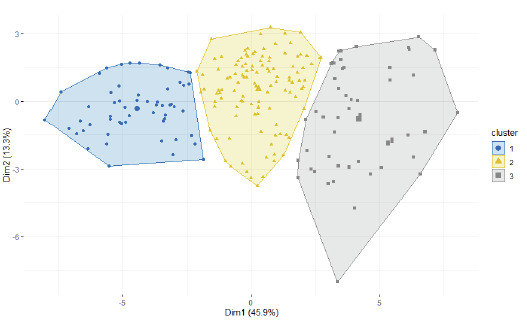
Plot of clusters from hierarchical clustering on principal components

Hierachical clustering performed based on principal components grouped the studied cultivars into three clusters (Figure 3). Clusters 1, 2 and 3 contained 54, 114 and 52 cultivars, respectively. Cluster 3 gathered the best cultivars showing the highest content of vitamins and amino acids (Table 3). For instance, cultivars of Cluster 3 were twice as rich in glutamic acid as those of Cluster 1. Cultivars of Cluster 1 had the lowest levels of vitamins and amino acids. Cluster 2 had relatively moderate contents for all the vitamins and amino-acids compounds. However, cultivars in Cluster 2 were the most endowed in VB1, VB3 and cellulose.

**Figure 4:**
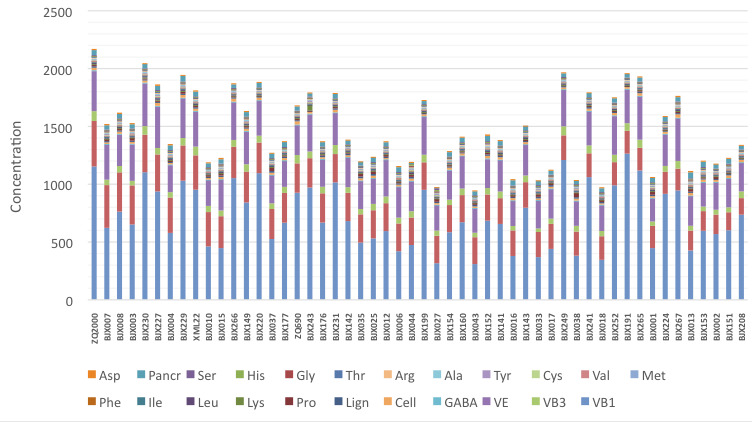
Stacked bar graph showing the performance of cultivars of cluster 3 based on the combination of vitamins, dietary fiber and amino-acids compounds. VB1, vitamin B1; VB2, vitamin B2; VB3, vitamin B3; VE, vitamin E; GABA, gamma-aminobutyric acid; Cell, cellulose; Lign, lignin; Pro, proline; Lys, lysine; Leu, leucine; Ile, isoleucine; Phe, phenylalanine; Met, methionine; Val, valine; Cys, cystine; Tyr, tyrosine; Ala, alanine; Arg, arginine; Thr, threonine; Gly, glycine; His, histidine; Ser, serine; Glu, glutamic acid and Asp, Aspartic acid.

The stacked bar graph presented on (Figure 4) was performed to identify within Cluster 3 a set of cultivars presenting the highest accumulation for all the analyzed compounds. Out of the 52 cultivars gathered in this Cluster, the best six are listed as follows: ZQ2000, BJX230, BJX229, BJX249, BJX191 and BJX265. They were mainly endowed in Glu, GABA, VB1, VB2 and VE.

## Discussion:

This study was designed to determine and evaluate the content of vitamins, dietary fibers, essential amino acids and non-essential amino acids in a wide collection of barley from Tibet. The approach used in this study was quantitative.

## Nutritional variability within the studied collection:

Thiamin (VB1), Ribolfavin (VB2), Niacin (VB3) and Tocopherol (VE) were diverse with coefficients of variation ranging from 15% to 35%. Referring to the scale of coefficients of variation proposed by Ouedraogo [30] and taken up by Kouyate [31] we deduce that there is a fairly significant variation in the vitamin content within our studied population. The vitamin content of barley grains varies widely. Un-germinated barley does not contain vitamins A, C and D, although the carotenoids and sterols that are present may act as precursors for vitamins A and D, respectively [32]. Vitamin E, a mixture of tocopherols, occurs in barley oil. Barley is unique among cereals having all eight naturally occurring tocopherols. The tocolpherols are found exclusively in germ tissue (embryo, scutellum) and tocotrienols in the starchy endosperm and aleurone [33]. Barley also contains B vitamins. These vitamins are mainly present in the embryo and the aleurone layer [34]. For amino acids, we noted the presence of all essential and non-essential amino acids in all cultivars. Leucine (7.4 ± 2.2 mg / g) was the most abundant of the essential amino acids with a coefficient of variation of 30% while methionine (0.5 ± 0.6 mg / g) represented the least abundant amino acid with a coefficient of 108% change. The average value of essential amino acids in this study was 27 mg / g. This value is lower than the values (32.99-38.75 mg / g) reported by Sterna et al.[35] for the essential amino acids of six barley varieties studied in Latvia. We could explain this difference by the large diversity within the studied population, with some cultivars having low levels of amino acids.

The major components of dietary fibers are arabinoxylan, arabinogalactan, cellulose, b-glucan, lignin, pentosan and resistant starch. These dietary fibers are often categorized into soluble and insoluble dietary fibers. Most of the part of b-glucan consisted of soluble dietary fiber. Cellulose and lignin are main dietary fibers found in barley, and are thought to limit the release of sugars and are responsible for reduced glycemic index [36,37].In this study, we determined cellulose and lignin contents, which are insoluble fibers and obtained approximately 30.9% content. The results obtained on dietary fibers are relatively higher than those reported in other studies. In particular, Anderson et al.[38] and Lahouar et al.[39] studied ten varieties of barley in the USA and four varieties of barley in Tunisia, respectively, and reported values close to 21% for the dietary fiber content. Hence, this study showed that our collection of barley could be considered as a good source of dietary fibers. Overall our nutritional survey of the 245 cultivars demonstrated that there is a great variability within the population, which could be exploited in barley improvement.

## Implication of clustering of the studied collection to the barley improvement for nutritional and functional properties:

The quantitative characterization in this study has essential implication in germplasm utilization of barley. Nutritionally enriched barley is always valuable for human diet and animal feeding. Therefore, it is essential to procure promising genetic resources to add in breeding programmes in order to develop high yielding cultivars with greater availability of vitamins, amino acids, carbohydrates, dietary fibers and microelements [40]. The genetic variability of each trait in the genotypes and relatedness of germplasm can be assessed by the clustering analysis [41]. Clustering analysis enables to identify the major traits with greater share and allows grouping of cultivars on the basis of similarities and dissimilarities. This helps in crop improvement with specific targeted trait of interest [42]. The hierarchical clustering analysis of our barley germplasm resulted in three clusters, each with specific nutritional characteristics. In particular, cultivars of Cluster 3 were the most nutritional ones, suggesting that they could be proposed and introduced in barley producing areas in Tibet region. However, cultivars from other clusters could also be very useful in developing cultivars with enhanced content of particular metabolites. For example, cultivars of Cluster 2 could be targeted for the development of cultivars with high VB1, VB3 and cellulose content. The disadvantage of high dietary fiber content is their ability to chelate some oligo-nutrients (minerals, vitamins) and thus, reduce their bioavailability to the body [43]. Then, breeding for cultivars with low levels of dietary fibers is the target of some breeding programmes [44,45]. In addition, barley is used for a wide range of applications such as in animal feeding. Barley grains enriched with protein, methionine, lysine, cysteine and tryptophan are highly suitable for ruminants [46]. Therefore, our study will facilitate the selection of cultivars to be used as parents in breeding programmes in order to meet the specific needs of each market. New genomics and molecular breeding technologies are allowing us to better understand and harness genetic variation. Useful genetic variation for barley breeding can be now connected to the assemble catalogue of gene sequences, facilitating the discovery of functional genes associated with key agronomic traits and increasing the precision and speed of barley improvement [47]. The genetic architecture of vitamins, amino acids and dietary fibers will be explored in future studies through quantitative trait loci (QTL) mapping or genome-wide association study [48].

## Conclusions:

The genetic variability with respect to nutritional content was evaluated in 245 cultivars of barley from Tibet region of china. Significant variation of the nutritional traits was observed and clustering analysis grouped the germplasm into three distinct clusters. The cultivars ZQ2000, BJX230, BJX229, BJX249, BJX191 and BJX265 had excellent nutritional contents. The study represents a solid foundation for future studies aiming at deciphering the genetic basis of natural variation of nutritional traits in barley.

## Figures and Tables

**Table 1:** Variability of the contents of vitamins, and dietary fibers within the barley cultivars collected in Tibet

	Mean± SD	Coefficient of variation (%)	Minimum	Maximum
Vitamins (mg kg^-1^)				
VB1	698.7±246.5	35	228.2	1327.4
VB2	230.2±50.6	22	82	427.7
VB3	55.3±10.4	19	30.4	89.5
VE	261.9±40.2	15	164.9	379.7
GABA	3.9±1.3	0.34	1.2	8.6
Dietary fibers (%)				
Cellulose	18.8±4.5	24	8.7	32.8
Lignin	12.1±2.5	21	5.4	30.6

**Table 2:** Variability of the contents of amino acids within the barley cultivars collected in Tibet

	Mean (mg/g)	Coefficient of variation (%)	Min	Max
Essential amino acids				
Leucine (Leu)	7.4±2.2	30	1	16.1
Valine (Val)	0.9±0.9	101	0.02	6.6
Threonine (Thr)	4.0±1.2	31	0.5	7.8
Phenylalanine (Phe)	5.5±1.9	34	0.3	12.6
Isoleucine (Ile)	3.8±1.2	31	0.5	8
Lysine (Lys)	3.9±3.2	82	1.3	49.4
Histidine (His)	1.2±1.9	163	0.02	19.6
Methionine (Met)	0.5±0.6	108	0.2	4.8
Σ^1^	27.2			
Non-essential amino acids				
Glutamic acid (Glu)	26.5±8.9	34	2.1	52.1
Aspartic acid (Asp)	5.9±2.4	41	0.7	12.4
γ-aminobutyric acid	3.9±1.3	34	1.2	8.6
Glycine (Gly)	4.6±1.6	34	0.1	8.7
Alanine (Ala)	4.6±1.5	33	0.7	8.7
Serine (Ser)	4.6±1.9	41	0.2	17.8
Proline (Pro)	9.8±8.2	83	0.8	64.9
Arginine (Arg)	5.6±2.1	36	0.6	12.6
Tyrosine (Tyr)	3.3±1.6	47	0.4	16.2
Cystine (Cys)	5.5±2.3	42	0.6	11
Σ^2^	74.3			
Σ	101.5			
Σ^1^, sum of the essential amino acids; Σ^2^, sum of the non-essential amino acids; Σ=Σ^1^+Σ^2^,

**Table 3:** Loadings of principal components for vitamin, dietary fibers and amino acids.

	Dim.1	Dim.2	Dim.3	Dim.4	Dim.5
Eigenvalue	11	3.2	1.86	1.23	1.09
Variance (%)	45.9	13.3	7.8	5.1	4.6
Cumulative variance (%)	45.9	59.2	66.9	72.1	76.6
					
Correlations between variables and axes					
VB1	0.35	0.82	0.16	0.04	-0.1
VB2	0.14	0.15	0.16	0.73	0.46
VB3	0.26	0.73	0.18	0.15	0.01
VE	0.39	0.7	0.22	0.2	0.09
GABA	0.73	0.08	0.01	0.13	0.07
Cell	0.14	0.52	0.03	-0.27	-0.14
Lign	-0.17	-0.22	-0.17	-0.2	0.22
Pro	0.17	-0.54	-0.1	0.26	-0.18
Lys	0.37	-0.01	0.63	-0.05	-0.48
Leu	0.93	-0.01	-0.16	-0.15	0.14
Ile	0.95	0.04	0.07	-0.11	-0.09
Phe	0.92	-0.17	-0.11	-0.15	0.12
Met	0.33	-0.59	0.37	0.29	-0.05
Val	0.53	-0.53	0.18	0.28	-0.04
Cys	0.89	0	-0.39	0.01	-0.04
Tyr	0.72	-0.17	0.61	-0.18	-0.03
Ala	0.94	0	-0.23	0.09	-0.08
Arg	0.96	-0.06	-0.01	-0.08	0.14
Thr	0.94	0.01	-0.03	-0.02	-0.04
Gly	0.89	-0.02	-0.36	0.05	-0.04
His	0.29	-0.3	0.5	-0.39	0.55
Ser	0.88	-0.09	0.18	0.01	-0.14
Glu	0.84	-0.05	-0.32	0.06	-0.18
Asp	0.75	0.22	-0.07	-0.1	0.3

**Table 4:** Characteristics of clusters from hierarchical clustering on principal components. Data are mean followed by standard error.

	Cluster 1	Cluster 2	Cluster 3	P-value
Number of cultivars	56	112	52	
VB1	536.12±32.06b	777.5±22.67a	720±33.27a	< 0.0001
VB2	226.7±6.99a	229.98±4.94a	243.24±7.26a	0.211
VB3	49.5±1.38b	57.92±0.98a	55.1±1.43a	< 0.0001
VE	240.27±5.21b	268.85±3.69a	274.09±5.41a	< 0.0001
GABA	3.01±0.13c	4.15±0.09b	5.24±0.14a	< 0.0001
Cell	17.55±0.59b	19.65±0.42a	18.23±0.62ab	0.01
Lign	12.58±0.33a	12.2±0.23a	11.49±0.34a	0.073
Pro	9.57±1.1b	8.21±0.78b	14.32±1.14a	< 0.0001
Lys	2.47±0.43c	3.88±0.3b	5.68±0.44a	< 0.0001
Leu	4.47±0.15c	7.67±0.11b	10.08±0.15a	< 0.0001
Ile	2.27±0.08c	3.9±0.06b	5.23±0.08a	< 0.0001
Phe	3.16±0.13c	5.63±0.09b	7.97±0.14a	< 0.0001
Met	0.44±0.07b	0.42±0.05b	0.98±0.07a	< 0.0001
Val	0.27±0.11c	0.88±0.08b	1.72±0.11a	< 0.0001
Cys	2.58±0.18c	5.82±0.13b	8.11±0.19a	< 0.0001
Tyr	1.83±0.16c	3.31±0.11b	4.93±0.17a	< 0.0001
Ala	2.72±0.11c	4.82±0.08b	6.44±0.11a	< 0.0001
Arg	3±0.13c	5.87±0.09b	8.35±0.14a	< 0.0001
Thr	2.33±0.08c	4.15±0.06b	5.5±0.09a	< 0.0001
Gly	2.79±0.12c	4.87±0.09b	6.47±0.13a	< 0.0001
His	0.58±0.25b	0.9±0.17b	2.28±0.26a	< 0.0001
Ser	2.45±0.14c	4.68±0.1b	6.54±0.14a	< 0.0001
Pancr	16.78±0.77c	26.7±0.55b	36.18±0.8a	< 0.0001
Asp	3.37±0.25c	6.16±0.18b	8.14±0.26a	< 0.0001
